# Percutaneous crossover rotational atherectomy recanalization: A case about health status and patency in a multimorbid vascular patient

**DOI:** 10.1016/j.ijscr.2024.110171

**Published:** 2024-08-13

**Authors:** Ahmed Algharib, Giel G. Koning

**Affiliations:** Department of Vascular & Endovascular Surgery, Euregio Hospital, Albert-Schweitzer-Straße 10, 48527 Nordhorn, Niedersachsen, Germany

**Keywords:** Recanalisation, Atherectomy, Rotational, Bycross, Crossover, Occlusion, Artery, Endovascular, Percutaneous, PAOD, Fontaine, Femoral, Bifurcation, Iliac, Health status, SF-36, Aspiration

## Abstract

**Introduction:**

Endovascular treatment of the common femoral artery (CFA) and its branches is often challenging. Sometimes, stent placement cannot be avoided. Furthermore, stent placement in this area carries several risks for complications. We present a challenging case in which we used a rotational atherectomy device percutaneously in cross-over-technique to recanalize the external iliac artery in combination with the femoral bifurcation, all in one session - and - without the need for a femoral stent in a multimorbid patient. We also tried to gain more insights in the patient's perspective and we took a validated health status evaluation into account.

**Presentation of case:**

The patient was presented due to chronic open wounds on the left foot for months (Stadium Fontaine IV). Duplex sonography and CT angiography showed a complete occlusion of the left external iliac artery with involvement of the left common femoral artery. Due to the pre-existing chronic diseases and the high risk of prolonged anesthesia, the patient was not suitable for open reconstruction of the common femoral artery. We aimed for endovascular therapy using a crossover maneuver to minimize anesthesia time as much as possible. The percutaneous treatment was performed with a rotational atherectomy device and drug-coated balloon angioplasty with satisfying angiographic results and complete blood-flow restoration. No peri-procedural complications occurred. We gained experience with this endovascular-treatment-device in our teaching hospital and more difficult cases can now be treated. The patient's perspective and health status were assessed during follow-up visit.

**Discussion:**

The endovascular treatment of severe calcifications in peripheral arterial occlusive disease (PAOD) seems to be a good solution for selected patients, significantly minimizing surgical trauma. The newly combined rotational atherectomy and thrombectomy devices have demonstrated positive outcomes in areas where conventional treatment has traditionally been the standard. The groin types of peripheral arterial occlusive disease (PAOD) are quite often challenging to operate. Open treatment of the common femoral artery has been the standard procedure until modern endovascular possibilities provide a new concept in this treatment, emphasizing a minimal invasive approach in multi morbid patients.

The case description results in an illustrated follow up period of 6 months and is presented in line with the recommendations of the consensus-based surgical case reporting guideline development.

**Conclusion:**

Managing peripheral arterial occlusive disease in the groin region poses a continual challenge. Traditionally, open treatment of the common femoral artery has been - and is - the established procedure. However, contemporary endovascular options now introduce a new paradigm in this treatment, highlighting minimally invasive approaches in multi morbid patients and its patient satisfaction.

## Highlights

• In this case we describe a complete percutaneous crossover revascularisation.
• Fontaine IV wound, without any healing trend was treated in a multimorbid vascular patient.
• The importance of this endovascular technique may contribute to the armamentarium of every (endo)vascular surgeon.

## 1 Introduction

Endovascular approach has popularized as treatment for peripheral arterial occlusive disease (PAOD), especially in the femoral-popliteal segment [1]. However, this concept mostly remains limited to the iliac-femoral artery. The dilation of the common femoral artery may be challenging without causing damage to one of the two branches [2]. Moreover, due to circumferential continuous calcification, the most negative influencing factor, a complete treatment without deploying a stent is not feasible. Overall, an open (conventional) treatment continues to be the standard in the area of the groin arteries. The concept of stent placement over a joint area, as is the area of the common femoral artery and the distal segment of the external iliac artery is, to date, not recommended. When nevertheless performed, potential complications such as re-stenosis, perforation, stent fracture and migration through the inguinal ligament, residual stenosis etcetera may very well occur. Due to these potential consequences, the endovascular approach, in this area, has been limited.

Using a rotational atherectomy device treatment in that area in combination with drug-coated balloons, without stent placement, is meanwhile possible [3]. The concept of this device combines two steps: debulking calcification (plaques) and thrombus through rotational atherectomy and thrombectomy, followed by aspiration. Subsequently, the target segments are dilated using a Drug-Coated-Balloon [4].

Presently, there are many endovascular devices to treat calcified plaques, such as rotational aspiration-atherectomy or orbital atherectomy, to debulk or destroy calcifications [4,5].

The concept of a combined system for debulking calcifications and aspirating thrombus is a novel adjunctive solution. It enables endovascular treatment in challenging areas that were previously only treated conventionally. In our opinion this may have a significant advantage, especially in older, multimorbid patients who may not tolerate long anesthesia times or complicated procedures (Level of Evidence 5 [6]). Furthermore, this allows for safe dilation without needing an extra filter system. Additionally, even after dilation, stent placement may be not required structurally [7]. This concept enables a complete endovascular treatment of the femoral arteries with a secure maneuver and technique.

Another important factor is, always, the evaluation of the health status of the patient after the procedure, in combination with the patient's perspective and experiences during follow-up.

The case description resulted in an illustrated follow up period of 6 months and is presented in line with the recommendations of the consensus-based surgical case reporting guideline development (SCARE) [8].

## 2 Presentation of case

A 61-year-old anemic man came with painful wounds in the left foot. He has a medical history of COPD (chronic obstructive pulmonary disease) GOLD stadium III, hepatic fibrosis (Child-Pugh-Stadium B), cholecystolithiasis, chronic renal insufficiency, Morbus Waldenström, Epilepsy, hypothyroidism, cardiac decompensation, coronary heart disease and PAOD (peripheral artery occlusive disease) Fontaine stadium IV on the left foot. The patient, who is a smoker with over 30 pack-years, falls under ASA-Class-IV (American Society of Anesthesiologists). All patient baseline characteristics are presented in the anemia was corrected during admission (Table 1 and Time Line).

**Table 1:** Laboratory findings at moment of admission.

Test	Value	International reference value
C-reactiveprotein (CRP)	300	<5 mg/L
Hemoglobin (Hb)	9.9	8.5–11.0 mmol/L
Red blood-cell count	4.29	4.3–6.0 × 10^12^/L
White blood-cell count	10.1	4.0–10.0 × 10^9^/L
Trombocytes	544	150–400 × 10^9^/L
Sodium	142	135–145 mmol/L
Potassium	3.75	3.5–5.0 mmol/L
Creatinine	69	50–110 μmol/L
Estimatedglomerularfiltration rate	102	>60 mL/min
Ureum	6.0	2.5–7.5 mmol/L

This multimorbid patient was presented due to open wounds on the left foot, which have been known for months. In physical examination a pulse of the Femoral common artery was not palpable. Clinically, there is compensated critical ischemia on the left. Vascular diagnostics revealed an ankle-brachial-index (ABI) of 0.3 on the left. Duplex sonography showed no flow signal over the left External Iliac Artery, with a proven occlusion of the left Common Femoral Artery. The Superficial Femoral Artery and the Profunda Femoris Artery displayed a marginal collateral flow, as did the Popliteal Artery and the crural arteries.

**Fig. 1:**
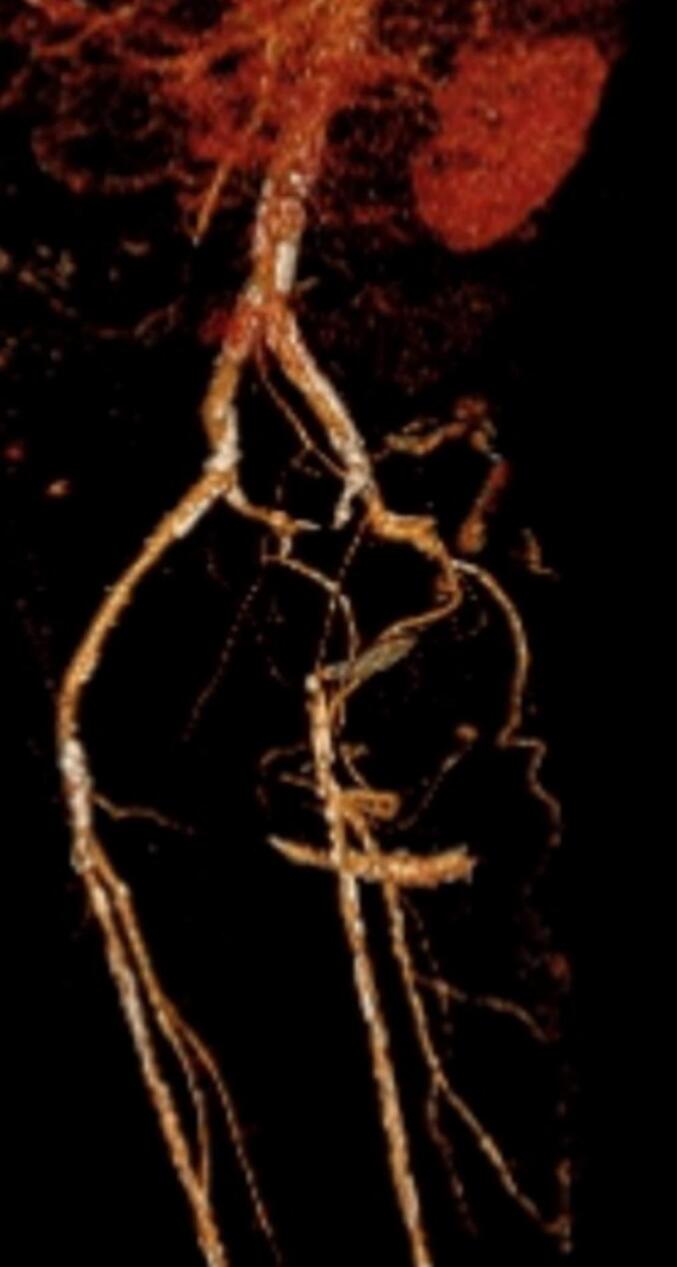
CT angiography showed a thrombo-embolic occlusion of the left external iliac artery and common femoral artery. However, the left superficial femoral artery and the left profunda femoris artery were visible from their origins.

The CT angiography revealed a mid-level stenosis of the Common Iliac Artery on the left side. The left External Iliac Artery and the left Common Femoral Artery were thrombo-embolic occluded with pronounced calcification. However, the left Superficial Femoral Artery and the left Profunda Femoris Artery were only visible from their origins (Fig. 1).

Due to the complex vascular lesions and in a multimorbid vascular patient, our approach was to initially pursue endovascular treatment to improve blood circulation in the feet for wound healing. Subsequently, in a second session and under loco-regional anesthesia, two more wound revisions (in separate sessions) were performed.

The procedure involved the following 10 steps:

1. Under sonographic guidance, the right Common Femoral Artery was punctured (Seldinger technique), a 6Fr sheath was positioned intraluminal, and the right iliac axis was successfully probed into the aorta.
2. Crossover probing of the left iliac axis; here, the stenosis in the mid-section of the left Common Iliac Artery was initially dilated with an 8 × 40 mm balloon (EverCross, Medtronic, Minneapolis, USA).
3. Placement of a crossoversheath 8Fr (from right CFA to left CIA) and probing of the occluded left iliac-femoral axis. Digital Substraction Angiography of the target vessel was performed after each critical step to check the intraluminal position.
4. Using the described rotational atherectomy device over 2 cycles over a semi-stiff wire 0.035 × 180 (ByCross® —6F— 95 cm, Taryag Medical Ltd. via: Plus Medica, Düsseldorf, Germany) (see also reference 4).
5. PTA of the CFA with Drug-Coated Balloons 6 × 80 mm and PTA of the EIA 7 × 60 mm (Admiral, Medtronic, Deggendorf, Germany) (Paclitaxel dose 3.5 μg/mm^2^).
6. Completion angiography demonstrated an entirely open External Iliac and Common Femoral Artery, with the Superficial Femoral and Profunda Femoris Arteries adequately visualized.
7. Crural DSA showed no signs of embolization nor occlusion after this intervention.
8. Due to residual stenose in the left Common Iliac Artery, stenting was unavoidable, performed with Balloon-Expandable Stent 8 × 37 mm (Visi-Pro, Medtronic, Minneapolis, USA).
9. Removal of the system with palpable pulses in the groin on both sides.
10. The right CFA wall puncture was closed by using Perclose ProStyle closure device (Abbott, Wiesbaden, Germany).

**Fig. 2:**
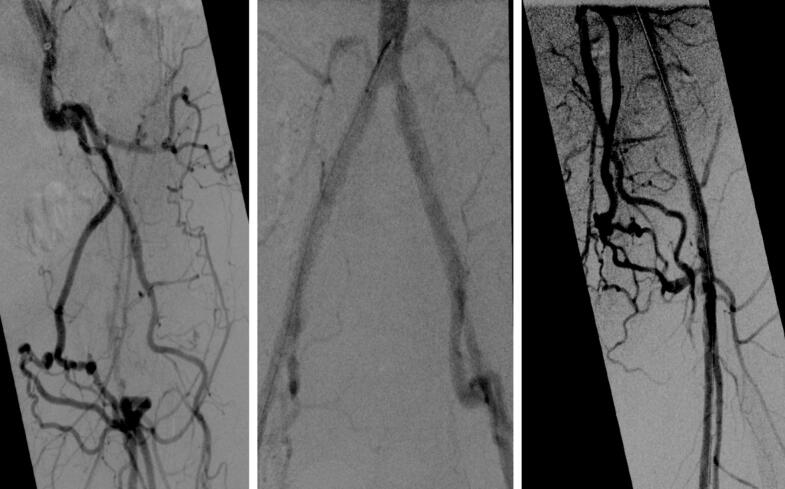
Angiography demonstrated occlusion of the left external iliac artery and common femoral artery. Postintervention showed a patent external iliac- and common femoral artery, with the superficial femoral and profunda femoris arteries adequately visualized.

**Fig. 3:**
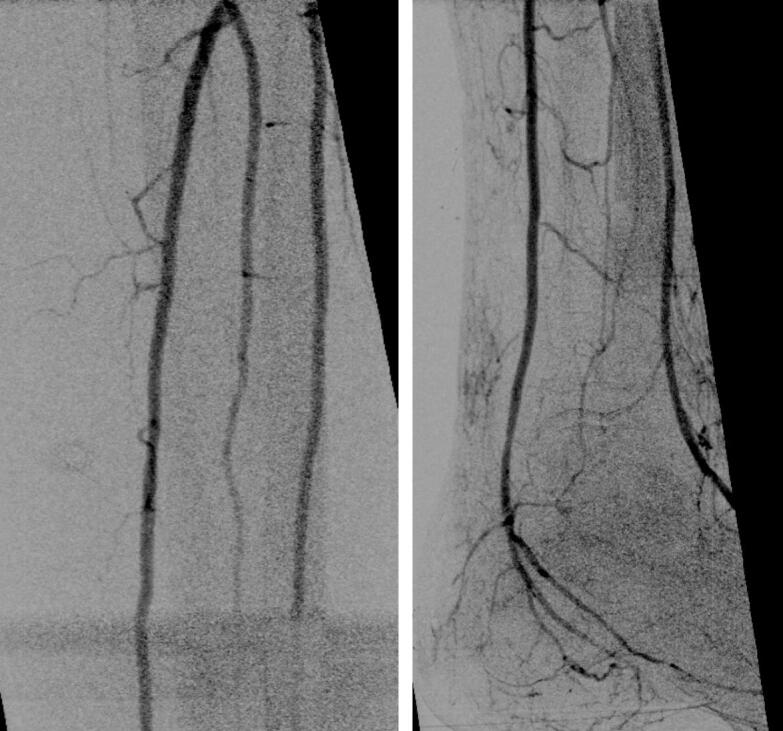
Completion angiography revealed no signs of embolism or peripheral occlusion following the procedure.

See also the Figs. 2 and 3.

Postoperatively, left feet were significantly better perfused, with no complaints of pain anymore, standard capillary refill time, ABI of 0.9 on the left treated side. The patient was satisfied. Duplex sonography revealed a patent left External Iliac Artery, the left Common Femoral Artery with a triphasic flow signal, and both the Superficial Femoral Artery and Profunda Femoris Artery on the left displaying triphasic flow. Following successful revascularization, a second session (on the fifth postoperative day) involved wound care in the form of revision, platelet-rich plasma (PRP) therapy [9], and fish skin cover (Kerecis, Lsafjörour, Iceland) [10]. On the 7th postoperative day, the patient could be discharged with closed wounds.

The German SF-36 form [14] evaluation revealed an overall ‘good’ general condition from the patient's perspective. He has no problems in the dimension: ‘activities of daily life’. He feels currently ‘better’ compared with the status of one year ago. He feels ‘not restricted nor impaired’ climbing the stairs.

He has ‘no mental problems’ and is ‘happy’. Sometimes he feels some ‘fatigue after a busy day’.

## 3 Discussion

Atherectomy serves as a method to address persistent and recurring arterial constrictions due to atherosclerosis, often seen in patients with peripheral arterial occlusive disease (PAOD). Utilizing a specialized catheter, this procedure targets the removal of adhering occlusive substances like plaques. Various configurations of atherectomy catheters exist, all designed for physical deposit removal. We chose the described device because it not only aspirates and crushes the removed substance but also continuously transports it out through the catheter. The procedure boasts low invasiveness, typically conducted via Femoral artery puncture and catheter introduction using the Seldinger technique.

This approach swiftly restores blood flow in narrowed or occluded vascular segments, particularly effective when employing athero-thrombectomy catheters for simultaneous clot and deposit removal. Moreover, the partial or complete removal of vascular deposits benefits adjunctive procedures like balloon dilatation (PTA) and stenting, potentially reducing complications and recurrences by minimizing the need to stretch the vascular wall. Although catheter-guided atherectomy has been debated for years, recent studies highlight the feasibility of multifunctional devices for complex lesions with moderate material consumption, offering an alternative to bypass surgery [11]. Comparison of the device's approval study results with data from various atherectomy systems sheds light on application scope, manufacturer-defined limitations, success rates, and complications. The 6-month follow-up and health status reveals a high revascularization rate, positioning the atherectomy device as a cost-effective alternative to bypass surgery, adaptable for both Iliac and Popliteal artery occlusions, yielding satisfactory outcomes [12].

Endovascular therapy for calcified vascular lesions represents a positive development in treating peripheral arterial occlusive disease (PAOD). Avoiding anesthesia and minimizing operative burden are significant advantages, especially for multimorbid patients.

This endovascular treatment can be integrated into hybrid procedures to combine the benefits of conventional therapy, which is an advancement. This helps shorten operation times and reduce patient trauma. The flexibility of the new endovascular devices with rotation atherectomy allows treatment in various areas [13]. This is a promising alternative and complement to conventional therapy for PAOD, improving patient care and expanding therapeutic options.

The most important factor for treatment of patients may very well be the evaluation of the opinion and the experience of the patient self. This always is a more or less subjective outcome measure. Therefore, we used as evaluation the German short form (SF-36) questionnaire after 3 and 6 months. This questionnaire is well known and validates in multiple health status studies for different surgical treatment outcomes [14]. Also, a costs analysis should be carried out as soon as we have a reliable sample size.

In our case, the patient was very ill with multiple pre-existing chronic diseases. A conventional treatment posed significant risks due to the extended anesthesia time and potential for additional wound complications. Moreover, the patient presented with open wounds on the left foot, partly infected, serving as potential infection foci for a fresh surgical incision. Therefore, the treatment with an endovascular concept involving only punctures proved to be the optimal solution. However, the challenge lay in treating occlusions in the external iliac artery and the common femoral artery endovascular. Treatment in these areas often involves stent placement, associated with several complications. We aimed to avoid this in the femoral artery, making the treatment with a rotational athero-thrombectomy device for a thromboembolic occlusion of the external iliac artery and common femoral artery the best approach. Stent placement was unnecessary in the femoral artery, and the common iliac artery was smoothly reestablished by using a stent (step 8) without complications [15]. A completion angiography at the end of the treatment revealed no signs of embolism or peripheral occlusion following the procedure.

**Fig. 4:**
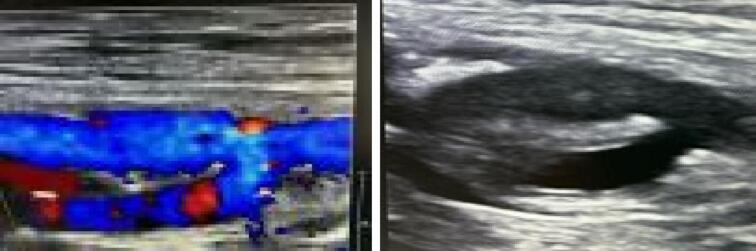
The duplex sonography control was performed at three and six months. The femoral axis remained consistently patent without signs of recurrent occlusion or stenosis throughout.

The duplex sonography control (Fig. 4) was performed directly postoperatively at three weeks, three and six months. The axis remained consistently patent without signs of recurrent occlusion or stenosis throughout. The ABI remained unchanged at 0.9 at six months follow up. In combination with the SF-36 results [14] the patient and his multidisciplinary team are satisfied. We think that the patient's opinion may very well be the most important outcome measure at the end of the day. To fulfill the patient's demand and to provide the best customized and patient-centered care we are convinced that a critical evaluation after care- and cure processes should be standardized in all vascular practices. A validated SF-36 (or other validated or more specific) evaluation as standardized exit- or discharge-talk could be recommended with in mind: ‘quality of life, what is it and who decides/defines it?’

This specific item nowadays, in the strong medical-technological era of vascular surgery, should not be forgotten. It requires more and more ‘soft skills’ from doctors to really assess the need of their patients and to fulfill most of the expectations of both sides in trust and management [16].

## 4 Conclusion

Managing peripheral arterial occlusive disease in the groin region poses a continual challenge. Traditionally, open treatment of the common femoral artery has been - and is - the established procedure. However, contemporary endovascular options now introduce a new paradigm in this treatment, highlighting minimally invasive approaches in multi morbid patients and its patient satisfaction.

## Consent

Written informed consent was obtained from the patient for publication of this case report and accompanying images. A copy of the written consent is available for review by the Editor-in-Chief of this journal on request.

## Ethical approval

Not required for this case report in line with permission of the Ärztekammer, Hannover, Germany.

Patient gave his full written permission to use data and photographs.

## Funding

None.

## Author contribution

AA: writing manuscript.

GGK: writing manuscript, critical reviewing, supervision manuscript preparation process.

## Guarantor

A. Algharib.

G.G. Koning.

## Research registration number

N/A.

## Conflict of interest statement

None.
